# Supplementary material for: Fascin1 empowers YAP mechanotransduction and promotes cholangiocarcinoma development
Source: Commun Biol. 2021 Jun 21;4:763. doi: 10.1038/s42003-021-02286-9 (PMC8217270; doi:10.1038/s42003-021-02286-9)
Supplement: Supplementary file 2 — Supplementary Information [file 42003_2021_2286_MOESM2_ESM.pdf]

Pocaterra et al., ***Fascin1 empowers YAP mechanotransduction and promotes cholangiocarcinoma development.***

Supplementary Information

Includes 4 Supplementary Figures and 2 Supplementary Tables

Supplementary Figure 1

a

Gene	Mean Ct value
GAPDH	23,8
FSCN1	22,9
FSCN2	39,6
FSCN3	44,9

b

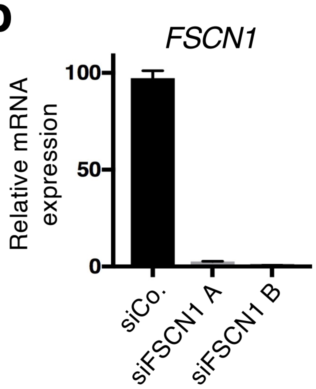

c

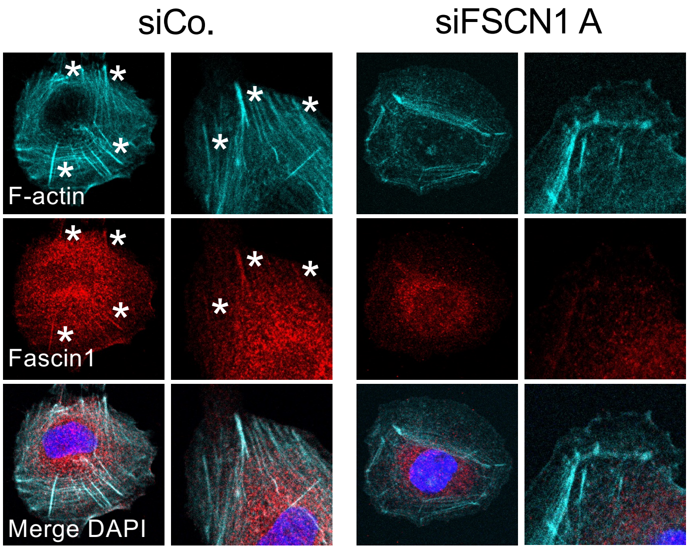

Supplementary Figure 1

a. Mean cycle threshold (Ct) values for the indicated mRNAs in MCF10A cells, based on the same cDNA dilution. *Fascin2 (FSCN2)* and *Fascin3* (*FSCN3)* are close to the specificity detection limit.

b. qPCR for *Fascin1 (FSCN1)* in MCF10A cells transfected with control siRNA (siCo.) or with two independent siRNAs targeting Fascin1 (siFascin1 A and siFascin1 B). Data are relative to *GAPDH* expression. Mean expression levels in the control sample were set to 100, and all other samples are expressed relative to this. Data are mean and s.d.

c. Representative immunofluorescence images of MCF10A cells transfected with FSCN1 siRNA (siFSCN1) or control siRNA (siCo.) and stained for Fascin1, F-actin (phalloidin) and DAPI. Asterisks indicate Fascin1-positive radial F-actin bundles. Scale bar= 10µm.

Supplementary Figure 2

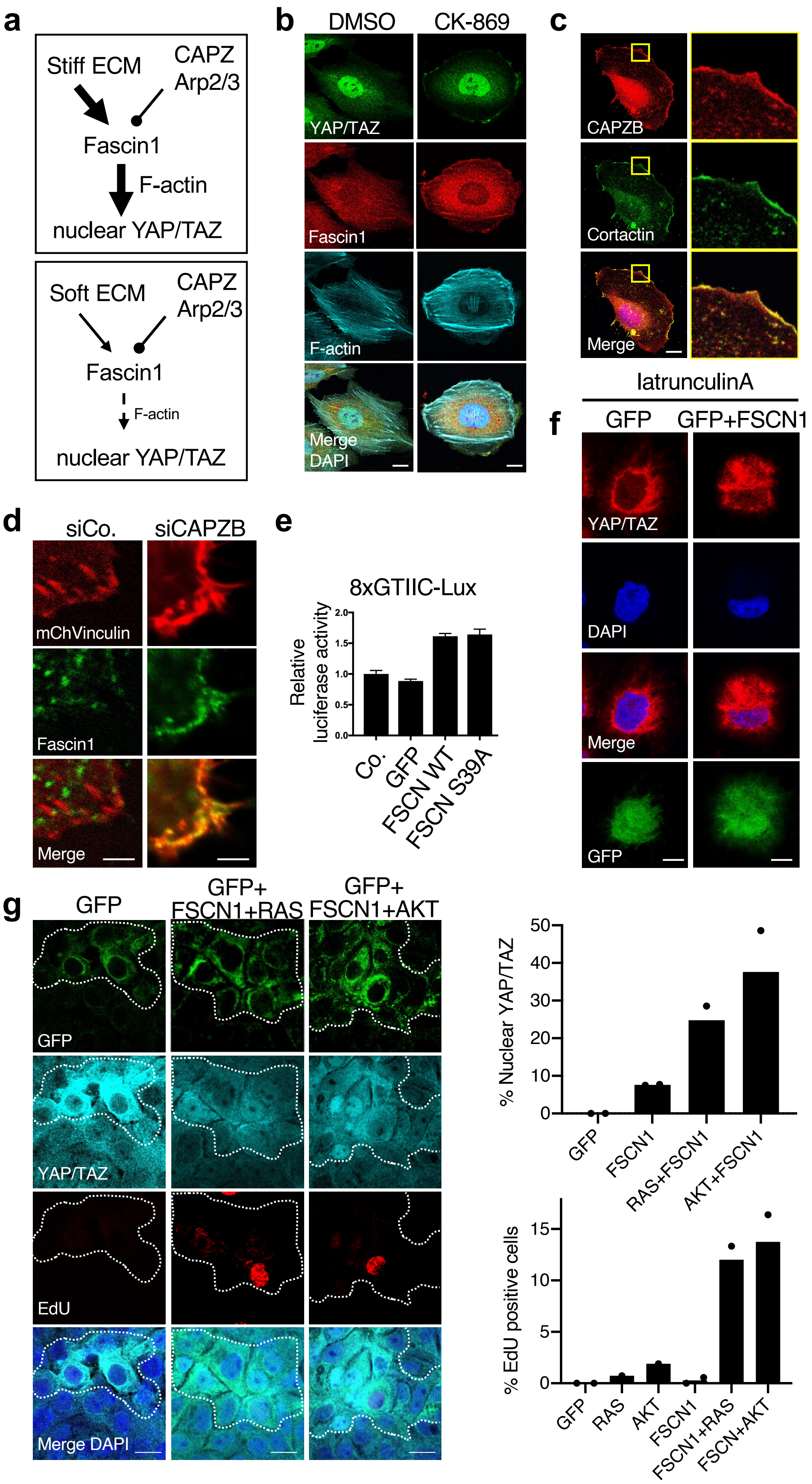

Supplementary Figure 2

a. A simplified scheme depicting the relationships between Fascin1, ECM stiffness and CAPZ-Arp2/3. A stiff ECM sustains the formation of a contractile cytoskeleton (F-actin), which requires Fascin1. In turn, this cytoskeleton promotes YAP/TAZ nuclear localization. In these conditions, the inhibitory effect of CAPZ and Arp2/3 is not strong enough to influence YAP/TAZ localization. On a soft ECM, formation of a contractile cytoskeleton is decreased, and it is competed by CAPZ and Arp2/3 activity, whose activity becomes relevant to regulate YAP/TAZ localization.

b. Representative immunofluorescence images of MCF10A cells treated on plastics with the CK-869 Arp2/3 inhibitor and vehicle control (DMSO) and stained for Fascin1, YAP/TAZ, F-actin (phalloidin) and DAPI as nuclear counterstain. Scale bar= 10μm.

c. Representative immunofluorescence images and magnifications (yellow insets) of MCF10A stained for endogenous CAPZB and Cortactin, used here as a marker of branched actin. Scale bar= 10μm.

d. Representative immunofluorescence images of MCF10A transfected with CAPZB siRNA (siCAPZB) or control siRNA (siCo.) together with plasmid expressing mCherry-Vinculin to visualize focal adhesions (FAs). Cells were then stained for endogenous Fascin1 to evaluate colocalization with mCherry-Vinculin. Scale bar = 1μm.

e. Luciferase assay with the 8XGTIIC-lux YAP/TAZ reporter in HEK293 cells at high density. Co. indicates cells transfected only with the reporters and filler DNA. Mean expression levels in the Co. sample were set to 1, and all other samples are expressed relative to this. n=2 experiments. Data are mean and s.d.

f. Representative immunofluorescence images of MCF10A cells transfected with GFP or with GFP+FSCN1 plasmids and treated with latrunculinA for 6 hours. Scale bar= 4μm

g. Representative immunofluorescence images of MCF10A cells transiently transfected with the indicated combinations of plasmids encoding for GFP, FSCN1, H-RAS G12V, and myristoylated AKT were seeded together with non-transfected cells at high density. After 48 hours, cells were harvested for analysis of YAP/TAZ localization and EdU incorporation (quantifications on the right). Dotted white lines indicate transfected cells. YAP/TAZ and EdU levels were similar in GFP and in surrounding non-transfected cells. Data are mean and single points. n=2 experiments.

Supplementary Figure 3

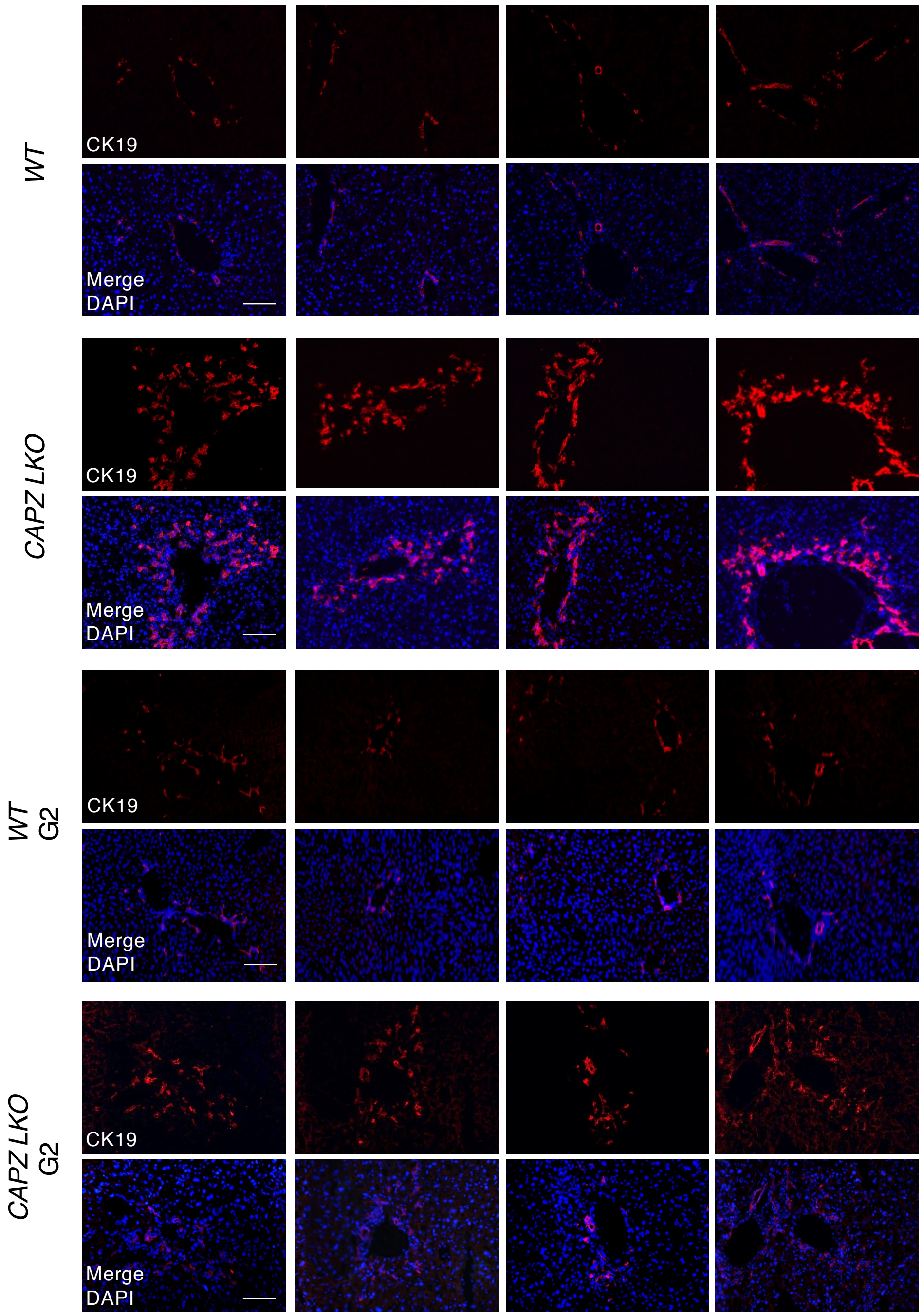

Supplementary Figure 3

Representative immunofluorescence stainings of liver sections from adult tamoxifen-injected Albumin-CreERT2; Capzbfl/fl mice (CAPZ LKO) mice injected i.p. with the G2 Fascin inhibitor or with vehicle (5% DMSO), stained for the cholangiocellular marker CK19 and DAPI as nuclear counterstain (quantifications in Figure 3a). Analyses were carried out 15 days after tamoxifen injection. Scale bar= 100µm.

Supplementary Figure 4

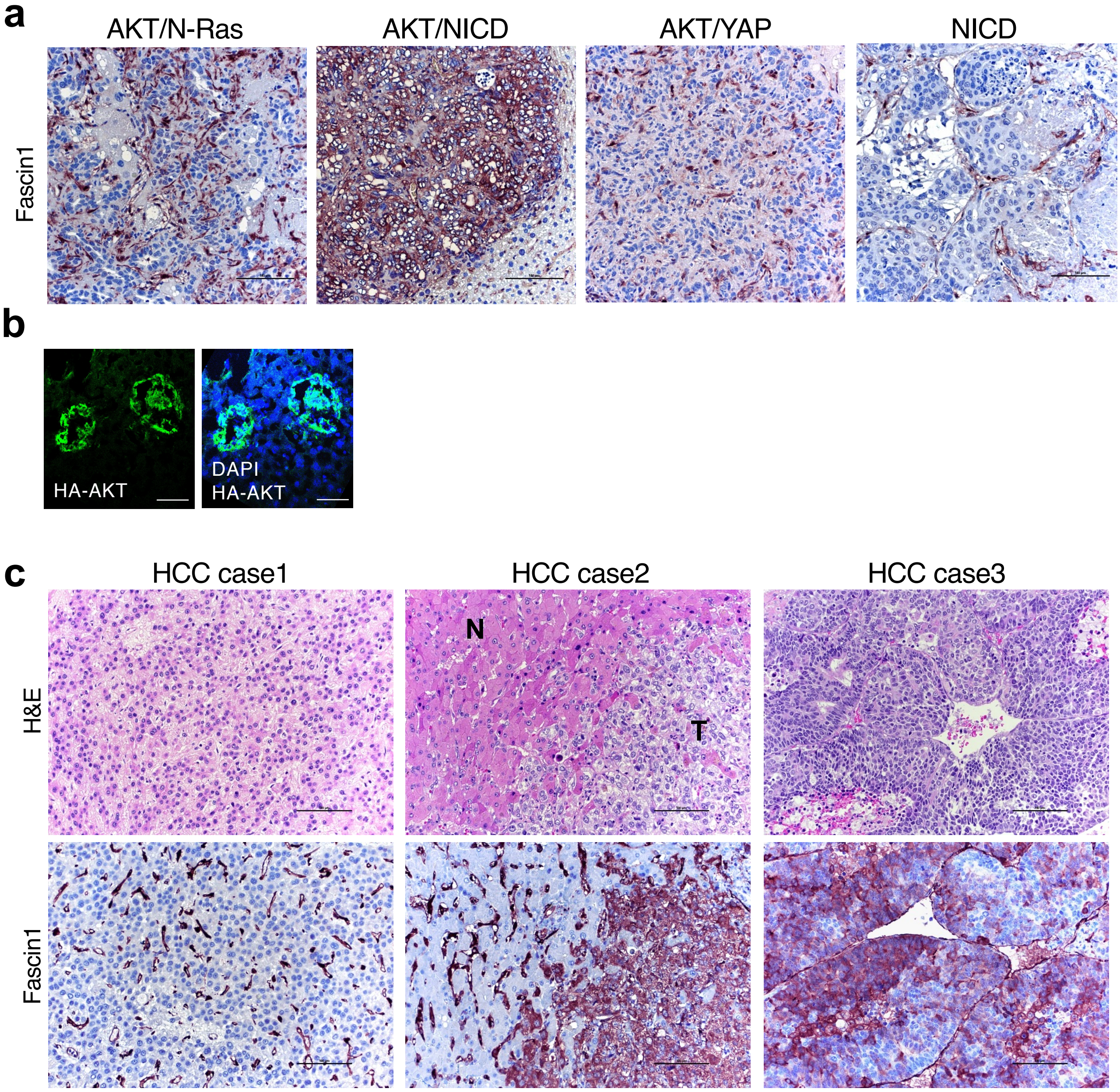

Supplementary Figure 4

a. Higher magnifications of immunohistochemistry images shown in Figure 4a. Original magnifications: 200x; scale bar= 100µm.

b. Representative immunofluorescence images of liver sections from C57BL/6N mice transduced by hydrodynamic tail vein (HTV) injection with transposon plasmids expressing myristoylated AKT (HA-AKT), Notch Intracellular Domain (NICD) and control short-hairpin RNA (shCo.). Liver sections were stained for HA to localize neoplastic lesions and DAPI as nuclear counterstain. Livers were analyzed 3 weeks after HTV. Scale bar= 45 µm

c. Left: a case of human hepatocellular carcinoma (HCC) exhibiting immunoreactivity for Fascin1 limited to liver sinusoids and endothelial cells. Middle: a case of HCC showing intense cytoplasmic staining for Fascin1 in tumor cells (T), compared to the non-tumorous surrounding liver (N). Left: a case of HCC displaying patchy immunolabeling for Fascin1 in tumor cells. Original magnification: 200x; scale bar= 100µm.

Supplementary Table 1. Clinicopathological features of intrahepatic cholangiocarcinoma (iCCA) patients

Variables	
No. of patients	62
Male	38
Female	24
Age (years)	
<60	26
>60	36
Etiology	
HBV	12
HCV	6
Hepatolithiasis	4
PSC	6
NA	34
Liver cirrhosis	
Yes	21
No	41
Tumor differentiation	
Well	25
Moderately	23
Poorly	14
Tumor size (cm)	
<5	46
>5	16
Tumor number	
Single	44
Multiple	18
Prognosis	
Better	
(≥ 3 years)	27
Poorer	
(< 3 years)	35

Abbreviations: NA, not available; PSC, primary sclerosing cholangitis

Supplementary Table 2. Clinicopathological features of hepatocellular carcinoma (HCC) patients

Variables	
No. of patients	50
Male	32
Female	18
Age (years)	
<60	16
>60	34
Etiology	
HBV	18
HCV	12
Ethanol	12
NA	8
Liver cirrhosis	
Yes	35
No	15
Edmondson and Steiner grade	
II	21
III	18
IV	11
Tumor size (cm)	
<3	28
>3	22
Alpha-fetoprotein secretion	
< 300 ng/ml	20
Multiple	30
Prognosis	
Better	
(≥ 3 years)	22
Poorer	
(< 3 years)	28

Abbreviations: NA, not available
