# Supplementary material for: Fascin1 empowers YAP mechanotransduction and promotes cholangiocarcinoma development
Source: Commun Biol. 2021 Jun 21;4:763. doi: 10.1038/s42003-021-02286-9 (PMC8217270; doi:10.1038/s42003-021-02286-9)
Supplement: Supplementary file 3 — Description of Supplementary Files [file 42003_2021_2286_MOESM3_ESM.pdf]

Description of Additional Supplementary Files

File name: Supplementary Data 1

Description: This file contains the source data for the figures.
